# Current Status of the Use of Antibiotics and the Antimicrobial Resistance in the Chilean Salmon Farms

**DOI:** 10.3389/fmicb.2018.01284

**Published:** 2018-06-18

**Authors:** Claudio D. Miranda, Felix A. Godoy, Matthew R. Lee

**Affiliations:** ^1^Laboratorio de Patobiología Acuática, Departamento de Acuicultura, Universidad Católica del Norte, Coquimbo, Chile; ^2^Centro AquaPacífico, Coquimbo, Chile; ^3^Centro i~mar, Universidad de Los Lagos, Puerto Montt, Chile

**Keywords:** antibiotics, salmon farming, antimicrobial resistance, *Piscirickettsia salmonis*, Chile

## Abstract

The Chilean salmon industry has undergone a rapid development making the country the world’s second largest producer of farmed salmon, but this growth has been accompanied by an intensive use of antibiotics. This overuse has become so significant that Chilean salmon aquaculture currently has one of the highest rates of antibiotic consumption per ton of harvested fish in the world. This review has focused on discussing use of antibiotics and current status of scientific knowledge regarding to incidence of antimicrobial resistance and associated genes in the Chilean salmonid farms. Over recent years there has been a consistent increase in the amount of antimicrobials used by Chilean salmonid farms, from 143.2 tons in 2010 to 382.5 tons in 2016. During 2016, Chilean companies utilized approximately 0.53 kg of antibiotics per ton of harvested salmon, 363.4 tons (95%) were used in marine farms, and 19.1 tons (5%) in freshwater farms dedicated to smolt production. Florfenicol and oxytetracycline were by far the most frequently used antibiotics during 2016 (82.5 and 16.8%, respectively), mainly being used to treat *Piscirickettsia salmonis*, currently considered the main bacterial threat to this industry. However, the increasing development of this industry in Chile, as well as the intensive use of antimicrobials, has not been accompanied by the necessary scientific research needed to understand the impact of the intensive use of antibiotics in this industry. Over the last two decades several studies assessing antimicrobial resistance and the resistome in the freshwater and marine environment impacted by salmon farming have been conducted, but information on the ecological and environmental consequences of antibiotic use in fish farming is still scarce. In addition, studies reporting the antimicrobial susceptibility of bacterial pathogens, mainly *P. salmonis*, have been developed, but a high number of these studies were aimed at setting their epidemiological cut-off values. In conclusion, further studies are urgently required, mainly focused on understanding the evolution and epidemiology of resistance genes in Chilean salmonid farming, and to investigate the feasibility of a link between these genes among bacteria from salmonid farms and human and fish pathogens.

## Introduction

It is well known that many fisheries resources have been overexploited, and that many are currently depleted, and unable to support the global demand for seafood. In this context, world aquaculture is seen as a key industry in satisfying the growing demand for food for human consumption. Currently, aquaculture supplies more than 50% of all the seafood produced for human consumption, having increased production 20-fold between 1970 and 2010 (up from 2.6 to 60.4 million of tons per year) with a mean annual growth rate of 7.8% (Troell et al., 2014), resulting in the fastest growing food-production industry in the world (FAO, 2014).

Chile is the eighth largest producer of aquaculture products in the world, with the salmonids (Atlantic salmon *Salmo salar*, rainbow trout *Oncorhynchus mykiss*, and Coho salmon *Oncorhynchus kisutch* – in order of relevance) and blue mussels (*Mytilus chilensis*) as the principal products (FAO, 2014). Chilean salmon aquaculture has developed rapidly over the last three decades, making Chile the world’s second largest producer of salmon after Norway, producing more than 900 thousand tons in 2014 (SERNAPESCA, 2017a). However, this high productivity has been achieved by intensive farming, i.e., huge biomass grown at high densities of fish per unit of water volume, which has resulted in an increased susceptibility of fish to diseases caused by viruses, bacteria, fungi, and parasites (Quesada et al., 2013). Common intensive husbandry practices as well as management procedures on salmon farms, such as stripping of broodstock, handling, vaccination, crowding, grading, starvation, antimicrobial treatments as well as loading and transport can lead to an increased susceptibility to a wide range of diseases. These stressors can also lead to injury and the impaired performance of reared salmon, which are usually kept in crowded conditions which facilitate the transmission of infectious pathologies (Poppe et al., 2002; Håstein, 2004). Thus, over recent decades, this increase in productivity has been accompanied by an increased use of chemicals, mainly antibiotics, which are commonly used for prevention and treatment of bacterial disease in salmon farming (Miranda, 2012). Antimicrobials used in salmonid farming are mainly administered to the fish through medicated feed, thus there is significant potential for a large proportion of the drug to enter the environment via uneaten medicated feed in addition to through urinary and fecal excretion (Cravedi et al., 1987; Kemper, 2008). It has been demonstrated that a significant amount of oxytetracycline is released through leaching from uneaten feed (Capone et al., 1996) and losses from uneaten feed may increase during a disease outbreak, especially if the disease or the lower palatability of medicated feed results in a loss of appetite (Hustvedt et al., 1991). This leads to the accumulation of antibiotic residues in the aquatic environment especially in marine sediments, where they can persist for months, favoring the selection of resistant microorganisms and consequently affecting the natural microbial activity and biogeochemical processes (Hollis and Ahmed, 2014).

Traditionally, antibiotics have been widely used in aquaculture to prevent and treat bacterial diseases (Romero et al., 2012). Excessive use of antibiotic in aquaculture in many countries has caused problems and concerns due to the development and dissemination of bacterial resistance, food safety hazards and environmental issues (World Health Organization, 2016). However, despite the negative impact of the use of antibiotics, the role of antibiotic usage in aquaculture in the development of resistance and dissemination of antimicrobial resistance genes (ARG) is still poorly understood (Done et al., 2015). Evidence suggests that antibiotics also promote the selection and spread of a broad and diverse set of ARG that form the resistome, facilitating the horizontal transfer of these genes among different bacteria and posing a health risk when they are transferred to human pathogens.

In this context, antibiotic use by the Chilean aquaculture is a particular case worth studying, because as far as it is known and based on the data available, production in Chile has one of the highest rates of antibiotic consumption per ton harvested worldwide. This is even more relevant, considering that high amounts of antibiotics are discharged annually into the waters of Chilean Patagonia, a pristine area of high conservation value, which contains a mosaic of unique ecosystems and three World Biosphere Reserves.

Various reviews have addressed at least partially the issue of antibiotic use in Chilean salmon farming (Cabello, 2004, 2006; Burridge et al., 2010; Millanao et al., 2011; Miranda, 2012; Romero et al., 2012; Cabello et al., 2013, 2016), mainly focusing on the potential impacts on human health, but studies providing information on the environmental consequences of the use of antibiotics in Chilean salmonid farming are still scarce. This review is focused on the available knowledge, encompassing information on antibiotic utilization over the last decade in Chilean salmonid aquaculture and the available published studies concerning antibiotic resistance in the farm associated microbiota and fish bacterial pathogens.

## Use of Antibiotics in Chilean Salmon Aquaculture

Antibiotics are not only utilized in human medicine, but also worldwide in livestock to treat bacterial infections and/or to promote animal growth (Du and Liu, 2012). Despite the lack of information on antibiotic use in many countries, worldwide antibiotic usage has been estimated to be in the range of 100–200 thousand tons per year (Wise, 2002; Kümmerer, 2003), with about half of this amount being used for veterinary purposes (Sarmah et al., 2006). For example, in 2009 13,000 tons were used in animal production within the United States of America alone (FDA, 2009), whereas 382.5 tons were used by the Chilean salmon industry during 2016. These levels must be of concern if it is taken into account that most of them are poorly absorbed at the tissue level and then excreted, at levels of between 40 and 90%, into the environment via animal urine or feces (Kemper, 2008).

The amount of antibiotics used in aquaculture worldwide is very difficult to estimate as the different countries involved vary widely with respect to their registration systems, and for this reason in many cases information is unavailable or impossible to compare due to gaps in the data (Heuer et al., 2009; Romero et al., 2012). However, within countries that have a registration system, a large variation in antibiotic use has been reported. For example, while Norway uses 1 g per ton of salmon produced, Vietnam requires 700 g per ton of shrimp (Smith, 2008). In fact, shrimp cultured in Vietnam along with Chilean salmon farming, are examples of industries exhibiting the highest rates of aquaculture antibiotic consumption in the world (Van Boeckel et al., 2015).

Chile is the second largest producer of salmon, accounting for approximately one third of the global salmonid production, behind only by Norway, and ahead of Scotland and Canada (Ibieta et al., 2011; Asche et al., 2013). However, Chile has significantly higher rates of antibiotic consumption than the other three countries. The amount used to produce 1 ton of salmon in Chile between 2011 and 2015 was on average more than 1,500 times higher than in Norway (NORM/NORM-VET, 2016; SERNAPESCA, 2017b).

This is of significant concern considering that the geographic area used by Chile for salmon farming is 4 times smaller than that used by Norway (Buschmann et al., 2006). Despite the fact that Norwegian production of farmed salmonids has more than doubled between 2003 and 2014, the use of antibacterials in aquaculture there has decreased by half over the same period (Directorate of Fisheries, 2015). This low antibiotic consumption is mainly a consequence of the availability of highly effective vaccines against furunculosis and vibriosis pathologies, as well as the rapid implementation of efficient zoo-sanitary measures and a significant improvement in biosecurity policies such as zoning and the spatial re-arrangement of marine production sites to minimize the horizontal spread of infections (Midtlyng et al., 2011). Unlike Norway, the higher mortality in Chile is attributed to bacterial infections as opposed to viruses, particularly the intracellular pathogen *Piscirickettsia salmonis* which causes the highest mortality in the marine phase of the culture and for which there are currently no effective vaccines nor an efficient and reliable antibiotic therapy (Rozas and Enríquez, 2014).

Looking at the antibiotic per ton of harvested salmon, during the last four years (2013–2016), Chilean companies used annually on average 580 g of antibiotic per ton of harvested salmon, surpassing the average levels used during the period 2005–2012 (438 g of antibiotic per ton of harvested salmon). Over recent years a consistent increase in the amount of antimicrobials used by Chilean salmonid farms, from 143.2 tons in 2010 to 382.5 tons in 2016, has been observed (SERNAPESCA, 2017b). During 2016, Chilean companies utilized approximately 0.53 kg of antibiotic per ton of harvested salmon, surpassing the levels used during 2005 and 2006 (0.39 and 0.53 kg per ton of harvested salmon, respectively), just prior to the infectious salmon anemia virus outbreak and the subsequent collapse of Chilean farmed fish production (**Table 1**). This indicates that beyond the fluctuations in the use of antibiotic during the last decade, the levels of antibiotic use by the Chilean farming salmon are far from decreasing. Of the 382.5 tons of antibiotics used on Chilean salmon farms during 2016, 363.4 tons (95%) were used in marine farms, whereas only 19.1 tons (5%) were used in freshwater centers dedicated to smolt production. These large differences in the quantities used are explained by the amount of antibiotic used to treat the *P. salmonis* bacterium in marine environments (SERNAPESCA, 2017b).

**Table 1:** Antibiotic use in Chilean salmon industry (SERNAPESCA, 2011, 2017b).

Year	Antimicrobial use (tons)	Harvested fish (thousands of tons)	Ratio (kg per harvested ton)
2005	239.2	614.0	0.39
2006	343.8	647.6	0.53
2007	385.6	600.6	0.64
2008	325.6	630.6	0.52
2009	184.5	474.2	0.39
2010	143.2	466.9	0.31
2011	206.8	649.5	0.32
2012	337.9	826.9	0.41
2013	450.7	786.1	0.57
2014	563.2	955.2	0.59
2015	557.2	883.1	0.63
2016	382.5	727.8	0.53

**FIGURE 1:**
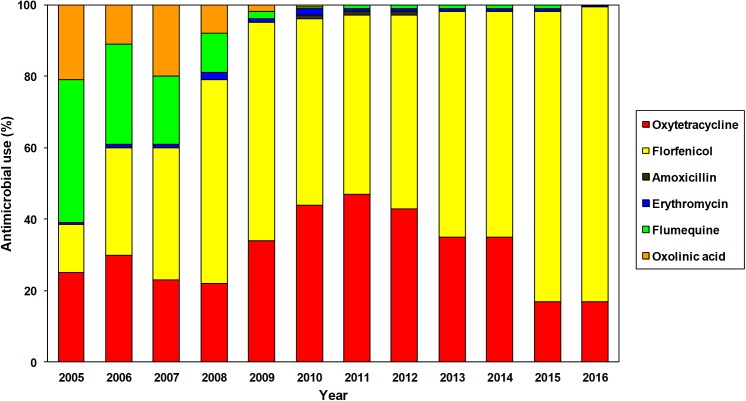
Annual use of antimicrobials authorized for use in Chilean salmon farming between 2005 and 2016 (SERNAPESCA, 2011, 2017b).

Among the six antibiotics currently approved for use in Chilean salmon aquaculture, florfenicol and oxytetracycline were by far the most frequently used during 2016 (82.5 and 16.8%, respectively) (SERNAPESCA, 2017b). It must be noted that the use of antibiotics has changed since 2005 (**Figure 1**), with an observable progressive increase in the use of florfenicol and oxytetracycline compared to the decrease in the use of the quinolones, oxolinic acid, and flumequine (SERNAPESCA, 2011, 2017b). The dominance of florfenicol in marine-based salmonid faming in Chile is mainly because it is the first choice for the treatment of *P. salmonis*, currently considered the main bacterial threat to the salmonid farm industry. The quinolones are a class of highly effective antibiotics extensively used in human medicine and consequently their use in animal production has been severely restricted by the World Health Organization, however, their use in animal production is not prohibited in many countries (Collignon et al., 2016). Despite the fact that during 2016 Chilean salmon farms did not report any use of oxolinic acid and that only 0.3% of the antimicrobials used was flumequine (**Figure 1**), it is clearly a priority to implement new regulations in the Chilean salmon industry, prohibiting the use of quinolones.

Despite the regulations and control of antibiotic usage in aquaculture imposed by the Chilean government, it must be concluded that until 2015 the use of antibiotics in this industry was higher than the amount reported. As an example only 22 out of 25 Chilean salmon farming companies agreed to release individualized information on their antimicrobial use in the marine phase of culture during 2015 (SERNAPESCA, 2016). To solve this issue, from 2016 it has been mandatory for all salmon companies in Chile to provide the information of their use of antibiotics during fish culture.

## Antibiotic Resistance in the Aquatic and Salmon Farm Environments

For many decades, the general opinion of scientists and physicians was that resistance to antibiotics and the presence of genetic determinants was a problem confined to the hospital environment. Only recently has it been recognized that antibiotic resistant microorganisms and associated resistance determinants are ubiquitous in nature, and that they are even present in pristine environments which have never been exposed to antimicrobial contamination (Allen et al., 2010; Knapp et al., 2011; Miranda, 2012). Several studies have indicated the occurrence of a great diversity of resistance genes, leading to the suggestion that the environment is a reservoir and an important source of new and emerging antibiotic resistance genes (ARGs) (Riesenfeld et al., 2004; D’Costa et al., 2006, 2007; Dantas et al., 2008; Allen et al., 2010; Donato et al., 2010; Wright, 2010). This discovery has led to a rethink on the origin of bacterial antibiotic resistance in pathogenic bacteria, accepting the assumption that the emergence of ARGs in pathogenic bacteria is likely to have arisen in natural environments (Nesme and Simonet, 2015). The term “resistome” was proposed in order to aid in our understanding of the origin, evolution and emergence of antibiotic resistance and was defined as the collection of all genes that might contribute to antimicrobial resistance (Wright, 2007). The resistome encompasses not only the genes encoding for antimicrobial resistance associated with bacterial pathogens, but also includes all the genes present in non-pathogenic species that dominate the natural environment (D’Costa et al., 2006). Thus, the resistome of a particular environment could include: precursor genes that express low resistance to antimicrobial molecules or affinity; cryptic resistance genes with no or low phenotypic expression in their host; and clinical resistance genes such as broad spectrum beta-lactamases, which confer resistance to high concentrations of antibiotics (Wright, 2007). It has been noted that ARGs present in pathogens can undertake different roles when they are found in an environmental host, as it is the host and the genomic context in which the gene is found that determines its phenotypic expression (Nesme and Simonet, 2015).

Traditionally, most of studies concerning antibiotic resistant bacteria and their resistance-encoding genes are based on techniques developed for cultivable bacteria, or molecular procedures using polymerase chain reaction primers only able to detect specific known antibiotic resistance-encoding genes (Miranda and Zemelman, 2002b; Buschmann et al., 2012; Di Cesare et al., 2013), but these techniques are unable to detect unknown ARGs (Petersen et al., 2002; Dang et al., 2008; Taviani et al., 2008). Furthermore, even when the use of these techniques has produced important findings, it has been concluded that they have the limitation of covering only a small fraction (< 0.1% in the marine environment) of the ARGs in the environment (Vaz-Moreira et al., 2014). The exponential increase in databases including sequences from genomes and metagenomes has allowed *in silico* sequence analysis of ARGs on the basis of comparisons with sequences described from pathogenic bacteria (Gibson et al., 2014; Nesme and Simonet, 2015). Functional metagenomics is a methodology that covers all components of a bacterial community (culturable and non-culturable) and does not depend on databases of previously known sequences which are generally isolated from bacteria from clinical settings (Mullany, 2014). Indeed, when genes with resistance phenotypes from metagenomic libraries are compared with known genes, frequently less than 65% of similarity at the amino-acid level is observed (Pehrsson et al., 2013). In a recent study using functional metagenomics on soil samples, nearly 3,000 genes encoding for antibiotic resistance were described, and most of them were new undescribed genes (Forsberg et al., 2014). Thus, different studies using functional metagenomics have found that ARGs are highly diverse and widely distributed, exhibiting little or no similarity to sequences of known genes (Lang et al., 2010; Schmieder and Edwards, 2012; Su et al., 2014).

The ARGs in natural ecosystems evolved over millions of years, long before the therapeutic use of antibiotics (Baquero et al., 2009). Currently, environmental resistomes are a vast and diverse collection of resistance genes, and also constitute a potential source of resistance genes for human pathogens (Martínez, 2008). There is significant evidence that various resistance genes present in human pathogenic bacteria have an environmental origin, strongly supporting the hypothesis that the transfer of genes encoding for antimicrobial resistance from the aquatic to the human clinic compartment is of importance.

However, until now it has been difficult to demonstrate the transfer of ARGs from the environment to clinically relevant bacteria or identify the mechanisms involved in this transfer (Finley et al., 2013; Perry and Wright, 2013; Vaz-Moreira et al., 2014). This may be due to the existence of restrictions or “bottlenecks” that modulate the transfer of resistance determinants from the original host to human pathogens, such as ecological connectivity, founder effects, and fitness costs as was noted by Martínez (2011).

The enhancement of selection and the environmental distribution of antibiotic resistant bacteria by the intensive use of antibiotics in aquaculture have been well-established (Smith, 2008; Miranda, 2012). Antibiotics used in fish aquaculture are typically administered via medicated feed, thus the first contact the antibiotic has with microorganisms occurs in the intestine of the fish. Considering the high densities of the bacterial populations present, the intestinal environment provides optimal conditions for the selection of antibiotic resistant bacteria (Le Bris et al., 2007). In fact, the increase in the levels of antibiotic resistant bacteria in the digestive system of fish under antimicrobial therapy is well documented (Austin and Al-Zahrani, 1988; DePaola et al., 1995). The next step is the dispersal of commensal or pathogenic antibiotic resistant bacteria from the intestinal environment to the water column or sediments through fish feces (Herwig et al., 1997; Samuelsen et al., 2000; Navarrete et al., 2008). It should also be considered that the medicated feed can also be ingested by wild fish living around the salmon cages, increasing the levels of antibiotic resistant bacteria in the intestine of these fishes also (Björklund et al., 1990; Ervik et al., 1994). Furthermore, the presence of antibiotic residues inside fish muscle has also been demonstrated, and obviously these residues can enter the human intestine if the fish is consumed without cooking (Fortt et al., 2007). The detection of tetracycline and quinolones in wild fish living near fish farms suggests that the environmental effects of antibiotic use in aquaculture have spread beyond the salmon farming cages (Fortt et al., 2007).

Marine sediments beneath fish cages are also an important compartment where selection of antibiotic resistant bacteria and the dissemination of the ARGs can be strongly enhanced. Many studies have demonstrated a strong correlation between the antibiotic use and the increase in antibiotic resistant bacteria in the sediments beneath the fish farm cages (Björklund et al., 1991; Herwig et al., 1997; Schmidt et al., 2000). In fact, bacteria resistant to antibiotics frequently administered in fish farms have been detected at high frequencies in fish farms and the surrounding aquatic environments (Nygaard et al., 1992; Samuelsen et al., 1992; Schmidt et al., 2000; Petersen et al., 2002; Cabello et al., 2016). Furthermore, the prophylactic and therapeutic utilization of antibiotics in aquaculture not only favors the selection of antibiotic resistant bacteria, but also the selection and dissemination of their respective antibiotic resistance-encoding genes (Yang et al., 2013). Consequently, genes codifying different resistances have been detected and quantified in fish farm environments (Tamminen et al., 2010; Muziasari et al., 2014). Similar results have been described for several tetracycline resistance genes [*tet(A)*, *tet(C)*, *tet(H)*, and *tet(M)*] (Tamminen et al., 2010). In another study, using one plasmid metagenomic library and high throughput sequencing, 58 genes codifying for resistance against 11 antibiotics were detected in marine sediments impacted by a fish farm (Yang et al., 2013). Many of these genes shared more than 90% similarity with transposons and plasmids described for human pathogens, suggesting the occurrence of an important frequency of mobility of these ARGs to human pathogenic bacteria (Yang et al., 2013). Another recent study performed on sediment samples from fish farms located in the Northern Baltic Sea, indicated that the resistome associated with fish farms can be from native ARGs enriched by antibiotic use, modifying the diversity and distribution of ARGs in the sediment (Muziasari et al., 2017). At the same time the enrichment of mobile genetic elements by antibiotic use was also detected, which indicates the potential risk of the ARGs spreading to other environments (Muziasari et al., 2017).

## Studies on Antibiotic Resistance Associated with Chilean Salmon Farming

### Farm-Associated Microbiota

Antibiotic use in aquaculture, as well as in other anthropogenic activities, has been widely associated with the selection and prevalence of resistant bacteria, and also the spread of their resistance genes (Cabello et al., 2013, 2016). This is something which must be of concern to the Chilean salmon industry, considering the large amounts of antibiotics used and the resulting high concentrations released into the surrounding aquatic environment (Kemper, 2008). Despite this concern, only a few studies concerning antimicrobial resistance in Chilean salmonid farming have been conducted in Chile (**Table 2**), and of these, only a few were related to the impact of this activity on the surrounding environment (**Table 3**). Among these, Buschmann et al. (2012) found barely measurable antibiotic concentrations, with the exception of flumequine, that was detected at trace levels in 8 of 36 collected sediment samples, with no significant differences between the control and impacted sites. The authors argued that presence of residues of flumequine in the sediment from an apparently pristine control site was probably the result of transport by water currents of both unchanged antimicrobials and their antimicrobially active metabolites, concluding that excessive use of antimicrobials in Chilean salmon aquaculture may also have an effect on marine sediments far from where these activities take place (Buschmann et al., 2012). Additionally, Contreras and Miranda (2011) detected no residues of oxytetracycline, florfenicol, flumequine, or oxolinic acid in sediments from eight salmon farms located in Southern Chile. Apparently, the persistence of antimicrobial residues in salmon farm impacted-sediments is higher at freshwater-based farms than in those below marine farms.

**Table 2:** Studies of antibacterial resistance in Chilean salmonid farming.

Issue	Number	Year
**Resistant microbiota**		
Freshwater	6	2002–2015
Marine	5	2012–2018
**Fish pathogens**		
*Piscirickettsia salmonis*	9	1996–2017
*Flavobacterium psychrophilum*	2	2012, 2016
*Aeromonas salmonicida*	1	2015
*Vibrio ordalii*	1	2013
*Streptococcus phocae*	1	2011

**Table 3:** Studies of antibiotic resistance of bacteria associated to Chilean salmonid farming.

Source		No. of isolates	Main result	Reference
Freshwater	Water, Pellet Sediment, Fish	103^A^	High proportions of low- and high-level OTC-resistant bacteria mainly from pellet and effluent samples. Resistant bacteria were mostly non-fermenting bacteria (77.7%), exhibiting MICs ranging from 64 to 2,048 μg mL^-1^.	Miranda and Zemelman, 2002b
	Water, Pellet Sediment, Fish	103^A^	A high number of bacteria resistant to AML, ERY, and FR, and an important frequency of resistance to FFC, CTX, and SXT was found, whereas resistance to G, K, FLU, and ENR was rather low. A high frequency (74 strains) of resistance to 6–10 antibacterial agents was detected.	Miranda and Zemelman, 2002a
	Water, Pellet Sediment, Fish	25^A^	Fifteen of the isolates carried one of seven different tetracycline (*tet*) genes [*tet(A)*, *tet(B)*, *tet(E)*, *tet(H)*, *tet(l)*, *tet(34)*, and *tet(35)*] and 10 had unknown *tet* genes	Miranda et al., 2003
	Water, Pellet Sediment, Fish	70^A^	Proportions of florfenicol resistance in under-cage sediments from salmon farm under florfenicol therapy (26.40%) were significantly higher than those from a farm with no recent history of antibacterial therapy (0.69%), detecting high levels of resistance to AML, ERY, FR, and SXT and susceptibility to G, K, and ENR	Miranda and Rojas, 2007
	Water, Pellet Sediment, Fish	119^A^	The *floR* gene was detected in 26 strains (21.8%) and most of the *floR*-carrying strains were glucose fermenters resistant to S and OTC. FFC resistance in most of non-fermenters (82 strains), was partially mediated by non-specific efflux pumps	Fernández-Alarcón et al., 2010
	Water, Pellet Sediment, Fish	10^A^	Six of the isolates carried the *tet(39)* gene, encoding for an efflux protein, such as the *Corynebacterium*, *Pseudomonas*, and *Psychrobacter* species.	Roberts et al., 2015
Seawater	Sediment	24^A^ + 24^C^	Increase of resistance to FFC, OT, and OA in aquaculture site. Detection of genes *Tet(A)*, *tet(B)*, *tet(S)*, *tet(K)*, *tet(M)*, *aac(6’)-Ib-cr*, and *intI1* among resistant isolates.	Buschmann et al., 2012
	Sediment	124^A^ + 76^C^	32, 16, and 53% of resistance to FFC, OT, and OA at aquaculture site. Detection of genes *tet(A)*, *tet(G)*, *dfrA1*, *dfrA5*, *dfrA13*, *sul1*, *sul2*, and *bla_TEM_* in resistant isolates.	Shah et al., 2014
	Sediment	4^A^	Isolates carried the *aac(6’)-Ib-cr* gene, conferring reduced susceptibility to quinolones and kanamycin.	Aedo et al., 2014
	Sediment Water	24^A^ + 24^C^	Genes *tet(A)*, *tet(B)*, *tet(K)*, *tet(M)*, *qnrA*, *qnrB*, *qnrS*, and *aac(6’)-Ib-cr* were detected in marine bacteria from aquaculture and control sites.	Tomova et al., 2015
	Sediment Water	23^A^ + 23^C^	*intI1* gene detected in isolates from aquaculture (11) and control (11) sites. *qnrA*, *qnrB*, and *qnrS* genes in four marine isolates were chromosomally located.	Tomova et al., 2018

*^A^, aquaculture impacted site; ^C^, control site; FFC, florfenicol; OT, oxytetracycline; OA, oxolinic acid; AML, amoxicillin; ERY, erythromycin; FR, furazolidone; CTX, cefotaxime; SXT, trimethoprim–sulfamethoxazole; G, gentamicin; K, kanamycin; FLU, flumequine; ENR, enrofloxacin; S, streptomycin.*

Based on the previous descriptions of the fate of antimicrobials in the aquatic environment, the lack of detection of highly persistent antimicrobials such as oxytetracycline, flumequine, and oxolinic acid in aquaculture impacted sediments, strongly suggests that these antimicrobials are mainly diluted and carried off by currents. In under-cage sediments, adsorption or attachment of antibiotics to particulate matter will usually result in their inactivation, but considering that these processes are dynamic and reversible, adsorbed antibiotics are expected to leach from these sites with their antibacterial activity intact and able to select for antimicrobial resistant bacteria, exerting a continuous low level selective pressure on the sedimentary microbiota. This could explain the recovery of high levels of antibiotic-resistant bacteria in under-cage sediments from farms with no history of antimicrobial usage, as was demonstrated by Miranda and Rojas (2007).

In Chile the detection and reporting of antimicrobial residues associated with the salmon farming industry is currently not mandatory. However, many salmon farming companies in Chile commonly carry out monitoring of various parameters, including assessments of sedimentary antibacterial residues from beneath salmon cages. Unfortunately this data is not made public nor is it made available to the Chilean regulatory agency. It is essential that the concentrations of antimicrobial residues in freshwater and marine sediments impacted by the Chilean salmonid industry are known in order that efficient guidelines for their regulation can be implemented. Currently only a veterinarian prescription is required to approve their use, and their progressive impact on the surrounding environment is not considered. It is strongly believed that the accumulation of antibacterial residues in sediments beneath salmon pens must preclude their use and that a rotation of the administered drugs is required.

It must be noted that even in the absence of detectable amounts of antimicrobials in water or sediments impacted by Chilean salmon farming, these environments are commonly associated with a high incidence of antibiotic multi-resistant bacteria and their respective resistance genes against a high diversity of antimicrobials, including oxytetracycline, florfenicol, and oxolinic acid (Miranda and Zemelman, 2002b; Miranda and Rojas, 2007; Buschmann et al., 2012). These results suggest that these environments enhance the persistence of resistant bacteria and associated genes even in absence of a selective pressure.

The most intensively used antibacterial in Chilean freshwater salmonid farms is oxytetracycline, comprising 86.8% of the total drugs used in freshwater-based farms for the treatment of flavobacteriosis during 2016 (SERNAPESCA, 2017b) and consequently various studies assessing the levels of oxytetracycline-resistant bacteria as well as characterizing their associated *tet* genes have been performed (Miranda and Zemelman, 2002a,b; Miranda et al., 2003; Roberts et al., 2015). Miranda and Zemelman (2002b) found a high proportion of bacterial resistance to high levels of oxytetracycline (100 μg mL^-1^) mainly from fingerling and effluent samples of a land-based farm (19.2 and 39.8%, respectively), as well as from the pelletized feed used in other salmon farms (34.3%). They found that resistant strains recovered from sampled farms showed high levels of resistance to oxytetracycline, exhibiting minimum inhibitory concentrations (MICs) ranging from 64 to 2,048 μg mL^-1^. Furthermore, Miranda and Zemelman (2002a) studied 103 oxytetracycline-resistant strains recovered from various sources at four Chilean freshwater salmonid farms, finding high taxonomic variability within the resistant microbiota, with a predominance of multi-drug resistant *Pseudomonas* strains. In addition, a high simultaneous resistance to various antimicrobials was detected in the studied strains, with 74 strains exhibiting resistance to 6–10 antimicrobials. Most of these strains showed resistance to amoxicillin, erythromycin and furazolidone, as well as a high frequency of resistance to florfenicol, cefotaxime, and trimethoprim–sulfamethoxazole, but a low incidence of resistance to quinolones.

In another study by Miranda and Rojas (2007) florfenicol resistance among microbiota associated with two Chilean freshwater-based salmon farms with different histories of antimicrobial usage and located in two different lakes was investigated providing evidence of high levels of resistance to florfenicol in under-cage sediments (26.4%) at the salmon farm with a recent history of florfenicol usage, whereas under-cage sediments at the salmon farm with no recent history of antimicrobial usage exhibited low levels of resistance (0.69%). However, it must be noted that non-impacted control sediments from one of the studied lakes also exhibited high levels of resistance (18.6%) with a high predominance of *Pseudomonas* species. The authors also observed the important occurrence of intrinsic resistance among resistant bacteria, as was observed by Kerry et al. (1994) for marine sediments free from anthropogenic impact, where a high incidence of pseudomonads, a group that exhibits innate resistance to various antimicrobials, was detected (Sengeløv et al., 2003). Finally, the use of unmedicated pelletized feed in a lake-based salmon farm was high (34.8%), suggesting that in certain cases this could be an important source of resistant bacteria for Chilean aquaculture impacted environments.

From these studies, an important number of resistant strains were demonstrated to carry several specific genes encoding for antimicrobial resistance such as *floR* and *tet* genes (Miranda et al., 2003; Fernández-Alarcón et al., 2010). In addition, a high number of other resistant strains were probably carrying new and previously uncharacterized antimicrobial-resistance encoding genes. This was recently demonstrated by Roberts et al. (2015), who studied 10 tetracycline-resistant strains isolated in 1999 from Chilean freshwater salmon farms, which tested negative for 22 *tet* genes, but six strains were later found to be carrying the *tet*(39) gene, while the other four strains most probably carried other unknown *tet* genes. To date, only two studies assessing the mobility of resistance encoding genes carried on bacteria recovered from various Chilean salmon farms have been conducted. Miranda et al. (2003) and Roberts et al. (2015) demonstrated the ability of a diverse group of *tet* genes to be transferred to an *Escherichia coli* recipient. This suggests that salmon farming is highly relevant to the enrichment of the environmental resistome, exhibiting the characteristics required to spread enteric bacterial species, which could play an important role in waterborne human disease. Despite the intensive use of large amounts of antimicrobials in the Chilean salmon farming industry and its role as an important reservoir of resistant bacteria carrying antibiotic-encoding genes, no studies of the transfer of genes encoding for antimicrobial resistance from salmon farming associated bacteria to pathogens have been conducted.

More recently, additional studies assessing antimicrobial resistance in the marine environment impacted by Chilean salmon farming have been conducted. In a study by Buschmann et al. (2012) strains recovered near to salmon culture cages in Chile exhibited high incidences of *tet*, *qnr*, and *floR* genes encoding for resistance to tetracyclines, quinolones and florfenicol, respectively, but in a later study the authors confirmed the absence of *qnr* and *floR* genes among these strains (Shah et al., 2014). In the most recent study, the authors found an important incidence of genes encoding for sulfonamide and trimethoprim resistance (*sul* and *dfrA*, respectively) as well as the presence of mobile genetic elements such as class 1 and 2 integrons (Shah et al., 2014). In addition, the same group identified the *aac(6)-Ib-cr* gene, encoding for an aminoglycoside acetyltransferase that confers reduced susceptibility to quinolone and kanamycin in marine bacteria associated with sediments impacted by a Chilean salmon farm, identical to the gene carried by urinary tract isolates of *E. coli*, suggesting the occurrence of a flow of this gene between these bacteria isolated from different environments (Aedo et al., 2014). In a more recent study, Tomova et al. (2015) studied a number of marine strains recovered from a Chilean aquaculture site at the same location, detecting in some of them the presence of *tet*, *qnr*, and *floR* genes, but concluding that undescribed tetracycline, quinolone and florfenicol resistance genes were probably carried by the majority of these strains. It must be noted that *qnr* genes encode for a low-level resistance to quinolones and are frequently associated with plasmids, suggesting a high feasibility of their mobility by horizontal transfer. Tomova et al. (2015) reported a high incidence of the *qnrB* gene among quinolone-selected bacteria and demonstrated that quinolone-resistant urinary *E. coli* isolated from patients living close to the sampled site were significantly enriched with *qnrB*, *qnrS*, and *qnrA* genes, compared to isolates from other regions not associated with aquaculture. The authors found that sequences of some of these genes were identical to those detected in the antimicrobial-resistant marine bacteria, and suggested the occurrence of horizontal gene transfer between antimicrobial-resistant marine bacteria and human pathogens (Tomova et al., 2015). Using the same isolates the authors detected the integrase encoding gene *intI1* in an important number of isolates recovered from non-impacted (11 isolates) and aquaculture impacted (11 isolates) sites (Tomova et al., 2018). Otherwise, the authors detected the chromosomally located *qnrA*, *qnrB*, and *qnrS* genes in four marine isolates, but these genes were no associated to integron gene cassettes (Tomova et al., 2018). In conclusion, these studies demonstrated a high concordance between the used antibiotics and the occurrence of associated resistance genes in Chilean salmonid farming providing evidence of an important occurrence of genes encoding for resistance to florfenicol (*floR*), tetracyclines (*tet*), and sulfonamides (*sul*), which suggest that this industry plays an important role as a reservoir of these genes.

Finally, it must be noted that all previous studies dealing with the issue of antimicrobial resistance in Chilean salmonid aquaculture have only considered the antibiotic resistant bacteria and some of the ARGs belonging to the culturable bacterial pool, which is known to be less than 1% of all environmental bacteria. Despite having proven that aquaculture supporting environments are an important source of new ARGs, the occurrence of important biases and limitations in our understanding of the real consequences of the release of these antibiotics into the aquatic environments must be recognized, and that an increased focus is required to demonstrate a direct relationship between environmental- and human-pathogenic antibiotic resistomes.

### Bacterial Pathogens

It should be noted that various studies reporting the antimicrobial resistance of several bacterial pathogens associated with Chilean salmon farms have been published (**Table 4**). In the absence of stated clinical breakpoints most of the studies of bacterial pathogens in Chilean aquaculture aim to generate standard protocols and establish epidemiological cut-off values to differentiate between wild-type (WT) and non-wild-type (NWT) populations. It must be noted that variations in cut-off values are indicative of changes in the antibiotic susceptibility of populations of the pathogenic species, but epidemiological cut-off (CO_WT_) values are protocol specific and need to be developed for all salmonid pathogens in Chile. Avendaño-Herrera et al. (2011) calculated the epidemiological cut-off values of florfenicol, erythromycin and oxytetracycline for *Streptococcus phocae* strains mostly recovered from diseased Atlantic salmon (*Salmo salar*), indicating that of the 19 strains isolated from 2004 onward, 18 strains were classified as NWT (non-fully susceptible). The authors suggested the importance of reducing oxytetracycline use for the streptococcal treatment. In another study, Henríquez-Núñez et al. (2012) studied a total of 40 *Flavobacterium psychrophilum* isolates obtained from Chilean salmon farms to determine their antimicrobial susceptibility to oxytetracycline, florfenicol, and oxolinic acid, finding that 90, 92.5, and 85%, respectively of strains were resistant to the three antimicrobials. Furthermore, 39 of the 40 isolates carried a single plasmid or combinations of two plasmids, but a relationship between plasmid and resistance could not be established. In a recent study, Miranda et al. (2016) determined the susceptibility of 125 *F. psychrophilum* Chilean isolates to antimicrobials used in fish farming and calculated their CO_WT_ values by using an agar dilution MIC method and a disk diffusion method. The data generated by the disk diffusion protocol used in this work were shown to have low precision, in agreement with Henríquez-Núñez et al. (2012), confirming that MIC determination would be the preferred method for susceptibility testing for this species. The NWT frequencies obtained using MIC data, were 24% for amoxicillin, 8% for florfenicol, and 70% for oxytetracycline, whereas for the quinolones oxolinic acid, flumequine, and enrofloxacin the frequencies of NWT isolates were 45, 39, and 38%, respectively using MIC data. The significant frequencies of isolates exhibiting reduced susceptibility to oxytetracycline and quinolones may result from treatment failures when these agents were used (Miranda et al., 2016). The occurrence of resistance to oxytetracycline, florfenicol, and oxolinic acid among some Chilean strains of *Vibrio ordalii* isolated from diseased salmonids has also been reported (Poblete-Morales et al., 2013). In a further study, Valdés et al. (2015) studied the draft genome sequence of an antibiotic-resistant strain of *Aeromonas salmonicida* isolated from infected rainbow trout, finding various efflux pumps and putative genes that confer resistance to macrolides, β-lactamics, florfenicol, and quinolones, concluding that efflux pumps are the main mechanisms of resistance to non-β-lactamic antibiotics.

**Table 4:** Studies of antibiotic resistance of pathogenic bacteria associated to Chilean salmonid farming.

Species	No. of isolates	Main result	Reference
*Piscirickettsia salmonis*	4	MIC and MBC values of CM, G, OTC, OA, and FLU using cytopathic effect on cell cultures	Smith et al., 1996
	2	A formulated medium is proposed to be used in antimicrobial susceptibility assays for *P. salmonis*	Yáñez et al., 2014
	20	Single point mutation in *gyrA* gene is responsible for the quinolone resistant phenotype	Henríquez et al., 2015
	292	ECO_WT_ values of FFC, OTC, OA, and FLU	Henríquez et al., 2016
	2	Florfenicol can modulate RND gene expression and increase efflux pump activity	Sandoval et al., 2016
	3 (genome)	Six specific genes, encoding for specific transporter proteins eventually relevant in conferring resistance to FFC and OTC	Cartes et al., 2017
	58	ECO_WT_ values of FFC and OTC using MIC data	Contreras-Lynch et al., 2017
	1 (genome)	The genome of an oxytetracycline-resistant strain bearing a multidrug-resistance plasmid is described	Bohle et al., 2017
	247	Resistance to quinolones (71.3%) and oxytetracycline (8.1%)	Saavedra et al., 2017
*Flavobacterium psychrophilum*	40	ECO_WT_ values of FFC, OTC, and OA for MIC data	Henríquez-Núñez et al., 2012
	125	ECO_WT_ values of AML, FFC, OTC, OA, FLU, and ENR using MIC and antibiogram data	Miranda et al., 2016
*Aeromonas salmonicida*	1 (genome)	Strain isolated from infected rainbow trout contained several efflux pumps and putative genes that confer resistance to macrolides, β-lactamics, florfenicol, and quinolones	Valdés et al., 2015
*Vibrio ordalii*	24	ECO_WT_ values of FFC, OTC and OA using MIC and antibiogram data	Poblete-Morales et al., 2013
*Streptococcus phocae*	31	ECO_WT_ values of ERY, FFC, and OTC	Avendaño-Herrera et al., 2011

*MIC, minimum inhibitory concentration; MBC, minimum bactericidal concentration; CM, chloramphenicol; G, gentamicin; AML, amoxicillin; FFC, florfenicol; OTC, oxytetracycline; OA, oxolinic acid; FLU, flumequine; ENR, enrofloxacin; ERY, erythromycin; RND, resistance nodulation division; ECO_WT_, epidemiological cut-off value for the fully susceptible to the antibacterial agent population (wild-type).*

Piscirickettsiosis, the disease caused by the intracellular pathogenic bacteria *P. salmonis*, is currently the most important bacterial pathology of seawater salmonid farming in Chile, accounting during 2016 for the 74.6 and 86.8% of the mortality in the Chilean salmon industry for Atlantic salmon and rainbow trout, respectively (SERNAPESCA, 2017a), and consequently it is the main target of antimicrobial therapies administered in the Chilean salmon industry (Rozas and Enríquez, 2014). With this in mind, based on a systematic review of available scientific literature, Mardones et al. (2018) concluded that the emergence and frequency of *P. salmonis* antibiotic resistant strains are topics which require further research, but the authors claimed that there is no published work that developed harmonized schemes for monitoring antimicrobial resistance and effectiveness against *P. salmonis*, neither the ecological impact nor costs associated with treatment strategies. However, various studies addressing the susceptibility to antimicrobial agents among Chilean *P. salmonis* strains have been conducted (**Table 4**). Smith et al. (1996) studied the antimicrobial susceptibility of four Chilean strains of *P. salmonis* by using cell monolayer-based MIC assays which detected significant variation in antimicrobial susceptibility patterns, whereas Yáñez et al. (2014) found a high susceptibility to florfenicol and oxytetracycline, but only three *P. salmonis* strains were studied. In another study, Henríquez et al. (2015) reported an important incidence of resistance to quinolones mediated by a single point mutation in the *gyrA* gene among *P. salmonis* strains isolated from diseased salmon in Chile. More recently, Henríquez et al. (2016) studied the susceptibility to quinolones, florfenicol, and oxytetracycline of 292 *P. salmonis* strains collected over 5 years, providing evidence of a high incidence of strains exhibiting resistance to quinolones, but suggesting that resistance to florfenicol and oxytetracycline is still developing. In further study, Sandoval et al. (2016) detected different florfenicol susceptibilities in two Chilean *P. salmonis* strains, observing that in the less susceptible strain florfenicol could modulate the gene expression of the multi-drug resistance-related efflux pump belonging to the resistance nodulation division (RND) family and increasing efflux pump activity. The authors concluded that the *acrAB* efflux pump is essential for *P. salmonis* survival at critical florfenicol concentrations and for the generation of antibiotic-resistant bacterial strains. More recently, Cartes et al. (2017) analyzed whole genomes of 3 *P. salmonis* isolates exhibiting different degrees of susceptibility to florfenicol and oxytetracycline, detecting genes encoding for specific transporter proteins. The authors suggested that these strains possess a greater number of membrane carriers, such as MDR (multidrug resistance) type (Cartes et al., 2017). On the other hand, Saavedra et al. (2017) studied a high number of isolates of this species, finding a high incidence of non-susceptible isolates to quinolones, but only a low percentage of non-susceptible to oxytetracycline, whereas all studied isolates were susceptible to florfenicol. In another recent study, Bohle et al. (2017) described the genome of an oxytetracycline-resistant *P. salmonis* isolate bearing a multidrug-resistance plasmid unique to this isolate and harboring a *tet* determinant, but no other resistance-encoding genes were described. Finally, in an attempt to standardize protocols and criteria for studying antibacterial susceptibility of this pathogen, Contreras-Lynch et al. (2017) proposed a standard protocol and stated the epidemiological cut-off values for florfenicol and oxytetracycline for this species.

## Conclusion

The growth of salmon aquaculture in Southern Chile is an example of industrial development that over only a few decades has gained a prominent place in global seafood markets. Along with this explosive development, this salmon farming industry has excessively utilized antibiotics to treat or prevent salmon diseases. Currently, 0.53 kg of antibiotics per ton of harvested salmon are used in the treatment and prevention of salmon diseases (data for 2016), 95% of which is used in the marine culturing phase to treat *P. salmonis* infections and 99.6% is comprised of just two antibiotics, florfenicol and oxytetracycline (SERNAPESCA, 2017b). Under this scenario, hundreds of tons of antibiotics enter the marine environment causing possibly negative environmental consequences and potential risks for human health. If we take account of the pharmacokinetic properties of both antibiotics, and assume that all administered antibiotic (by feed) was consumed, we can estimate that 40 tons of oxytetracycline and 3 tons of florfenicol were released into the marine environment in 2016. This is highly significant considering that in the last 10 years these antibiotics have been the most frequently used by industry.

Antibiotics entering marine environments favor the selection of antibiotic resistance among environmental bacteria and fish pathogens, and may also affect the activity of bacteria driving biogeochemical cycles in marine sediments. Furthermore, these chemicals can modify resistomes by selecting antibiotic resistance genes (ARGs) and increasing the rates of horizontal gene transfer, thereby increasing the probability of antibiotic resistance gene transfer from environmental to human pathogenic bacteria. These effects are of significant importance for Southern Chile, where antibiotics are used excessively in salmonid farming when compared to the other salmon producing countries. Therefore, antibiotic use by Chilean salmon farms has become a controversial issue due to the possible effects of high concentrations of antibiotics being released into nominally pristine environments, such as Chilean Patagonia. The Pacific coast of Patagonia is comprised of a vast area of fjords and canals, much of which is protected either within National Parks or close to them. Yet despite this protection the areas being used for aquaculture are constantly expanding into ever more remote and previously unimpacted areas.

Despite that over the last two decades only few studies assessing antimicrobial resistance and the resistome in the freshwater and marine environment impacted by salmon farming have been conducted, most of them demonstrated that Chilean salmonid farm industry plays an important role as a reservoir of antibiotic resistant microbiota and associated resistance genes. Furthermore, previous studies have shown that even in the absence of detectable amounts of antimicrobials in several sediments impacted by Chilean salmon farming, these environments are commonly associated with a high incidence of antibiotic multi-resistant bacteria and their respective resistance genes against a high diversity of antimicrobials, including oxytetracycline, florfenicol, and oxolinic acid. This might suggest that these environments enhance the selection and persistence of resistant bacteria and associated genes even in the absence of a selective pressure.

Considering that the Chilean salmon farming industry is one of the worldwide leaders in the use of antibiotics, studies on antibiotic resistant microbiota and related resistome are still very scarce and much data is required to understand the role of these environments in the maintenance and dissemination of antibacterial resistance. Thus, studies aimed at increasing knowledge of environmental resistomes associated with Chilean salmon farming and the possibility of their mobilization to the human clinical compartment are crucial for managing the potential threat to human public health. In this trend, surveillance studies of antibacterial resistance in under-cage sediments must be mandatory for all Chilean salmonid farms to avoid spread of selected resistance/genes to the human clinical compartment.

Furthermore, the growing incidence of antimicrobial-resistance among bacterial pathogens causing outbreaks in the Chilean salmon industry is probably a consequence of the intensive use of antibiotics in this industry, suggesting the urgent requirement for the application of a strict controls in order to avoid the overuse of antimicrobials, and the implementation of a regular surveillance program in order to detect the emergence and prevalence of ARGs in the environment. The observed irregular effects of antimicrobial therapies in controlling *P. salmonis* in Chilean salmonid farms suggest that the bacterium has developed some level of resistance. Thus, it is important that the rational and well-controlled use of antimicrobials is implemented soon in order to decrease the selective pressure imposed on this pathogenic species and consequently avoid the selection of multi-drug resistant strains.

In conclusion, further studies are urgently required, mainly focused on understanding the evolution and epidemiology of resistance genes in Chilean salmonid farming, particularly those encoding for resistance to antibiotics used in humans and to determine the feasibility of a link between these genes among bacteria from salmonid farms and human and fish pathogens. Furthermore, a harmonization of protocols and epidemiological cut-off values used to categorize pathogen isolates in all diagnostic labs is urgently required to avoid therapy failures. Considering that *P. salmonis* is a particularly important pathogen in Chilean salmon farming, causing the highest mortalities from infectious diseases (SERNAPESCA, 2017a), the development of efficient strategies for its control as well as understanding its antimicrobial susceptibility status, should be an urgent priority for the industry. Because of this trend, is understandable that most of published studies are related to this pathogenic species. Various Chilean researchers are currently elucidating the antibacterial resistance mechanisms involved in detected non-susceptible isolates, in accordance with the conclusions stated in a recent study (Mardones et al., 2018). Finally, having demonstrated the high prevalence of antibiotic resistant bacteria carrying transferable resistance genes in the Chilean salmonid farm industry it is an urgent necessity to implement antibiotic resistance surveillance programs and a high number of complementary initiatives to reduce the rate of increase of resistance in this industry. It is important to note that dissemination of surveillance data should not be restricted to the scientific community but must include all major stakeholders including the Chilean government regulatory agencies.

## Author Contributions

CM and FG conceived the review outline, researched and drafted the manuscript, and are the corresponding authors and primary contacts during the manuscript submission, review, and publication process. ML contributed significantly to the drafting and revisions of the manuscript. All authors have made intellectual contribution to the work, and approved it for publication.

## Conflict of Interest Statement

The authors declare that the research was conducted in the absence of any commercial or financial relationships that could be construed as a potential conflict of interest.
